# Genome Sequence of a Weissella confusa Strain Isolated from the First Reported Case of Neonatal Sepsis in an Equid

**DOI:** 10.1128/MRA.00066-20

**Published:** 2020-02-27

**Authors:** Sara V. Little, Andrew E. Hillhouse, Sara D. Lawhon

**Affiliations:** a Department of Veterinary Pathobiology, College of Veterinary Medicine & Biomedical Sciences, Texas A&M University, College Station, Texas, USA; b Texas A&M Institute for Genome Sciences and Society, College of Veterinary Medicine & Biomedical Sciences, Texas A&M University, College Station, Texas, USA; University of Maryland School of Medicine

## Abstract

The genome of a Weissella confusa strain isolated from a foal with sepsis is reported. Weissella confusa inhabits feces and causes disease in immunocompromised humans and animals. It is important for veterinarians to be aware of the pathogenic ability of these bacteria due to the unknown potential for zoonotic transmission.

## ANNOUNCEMENT

Weissella confusa, previously Lactobacillus confusus, is a Gram-positive coccobacillus that normally inhabits feces and can cause septicemia and endocarditis in immunocompromised people (1–7). Here, we present the genome of an isolate cultured from the blood of a foal with sepsis in the first reported case of such infection in an equid (8).

For all of the following procedures, default parameters and manufacturer’s protocols were used unless otherwise stated.

The blood was inoculated into a commercial blood culture system (Bactec Plus Aerobic/F culture vials; BD, Franklin Lakes, NJ). At 24 h, 48 h, and 7 days, an aliquot of the broth from the bottle was inoculated onto Trypticase soy agar supplemented with 5% sheep blood (blood agar plate [BAP]). The organism grew on plates within 24 h. Initial identification of the organism was made using sequencing of the 16S ribosomal DNA as previously described (8). Isolates were stored at –80°C in Brucella broth supplemented with 10% glycerol and revived for sequencing by inoculating an aliquot of the frozen bacteria onto a BAP. An isolated colony was used to inoculate a 5-ml culture of Trypticase soy broth which was incubated overnight. All cultures were incubated at 35°C ± 2°C in an atmosphere supplemented with 5% CO_2_. Next, 1-ml aliquots of each isolate were pelleted and subsequently lysed in a Qiagen TissueLyser using Macherey-Nagel bead tubes (type B) and lysis buffer from the NucleoMag tissue DNA kit. DNA extraction was performed via the manufacturer’s protocol (Macherey-Nagel).

The quality of genomic DNA was verified using the genomic DNA TapeStation (Agilent). Illumina libraries were prepared using the Illumina Nextera DNA Flex library preparation kit. Sequencing was performed using an Illumina MiSeq V3 2 × 300-bp kit. The resultant data were uploaded onto Illumina’s BaseSpace for all run monitoring, FASTQ generation, demultiplexing, and adapter trimming.

The sequencing output of the paired-end read sets contained 2,871,880 reads of 301 bp for approximately 390× coverage. The reads were assembled using SPAdes version 3.13.0 with the parameter –careful (9). The final assembly contained 151 contigs and a total of 2,239,284 bp with a GC content of 44.88%. The largest contig was 289,449 bp. The genome was analyzed for completeness using the *Firmicutes* database in BUSCO with a resultant score of 97%. Species identification was confirmed using ribosomal multilocus sequence typing (rMLST) with 100% support (10).

### Data availability.

This whole-genome shotgun project has been deposited at DDBJ/ENA/GenBank under the accession number JAAAMQ000000000. The version described in this paper is the first version. The raw MiSeq reads are available under SRA accession number SRR10850301.
